# Supplementary material for: Golden section criterion to achieve droplet trampoline effect on metal-based superhydrophobic surface
Source: Nat Commun. 2023 Oct 18;14:6572. doi: 10.1038/s41467-023-42375-3 (PMC10584815; doi:10.1038/s41467-023-42375-3)
Supplement: Supplementary file 1 — Supplementary Information [file 41467_2023_42375_MOESM1_ESM.pdf]

Golden section criterion to achieve droplet trampoline effect on metal-based superhydrophobic surface

Shengteng Zhao [1], Zhichao Ma [1, 2, 3, 4 *], Mingkai Song [1], Libo Tan [1], Hongwei Zhao [1, 3, 4] and Luquan Ren [2, 4, 5]

[1] School of Mechanical and Aerospace Engineering, Jilin University, Changchun, 130025, China

[2] Key Laboratory of Bionic Engineering Ministry of Education, Jilin University, Changchun, 130025, China

[3] Key Laboratory of CNC Equipment Reliability, Ministry of Education, Jilin University, Changchun, 130025, China

[4] Institute of Structured and Architected Materials, Liaoning Academy of Materials, Shenyang 110167, China.

[5] Weihai Institute for Bionics-Jilin University, Weihai, 264207, China

[*] E-mail address of the corresponding author: zcma@jlu.edu.cn (Zhichao Ma)

Supplementary Figures

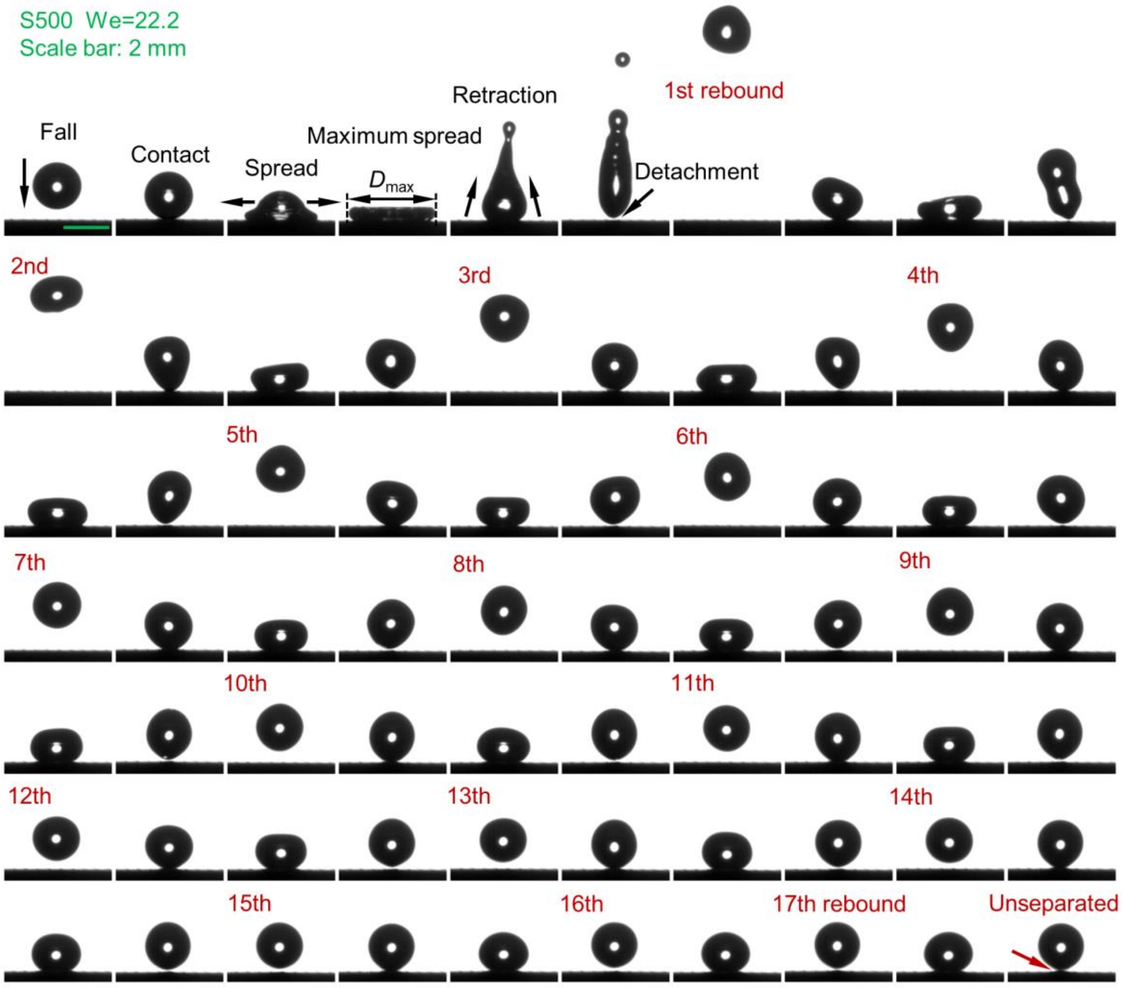

Supplementary Figure 1 | Seventeen times of droplet consecutive rebounds at the *We* of 22.22 on S500 surface. A complete droplet rebound cycle consists of four stages: fall, spread, retraction and rise. The kinetic energy of the droplet was gradually lost in each complete rebound process until the residual kinetic energy was insufficient to support the droplet to separate from the surface.

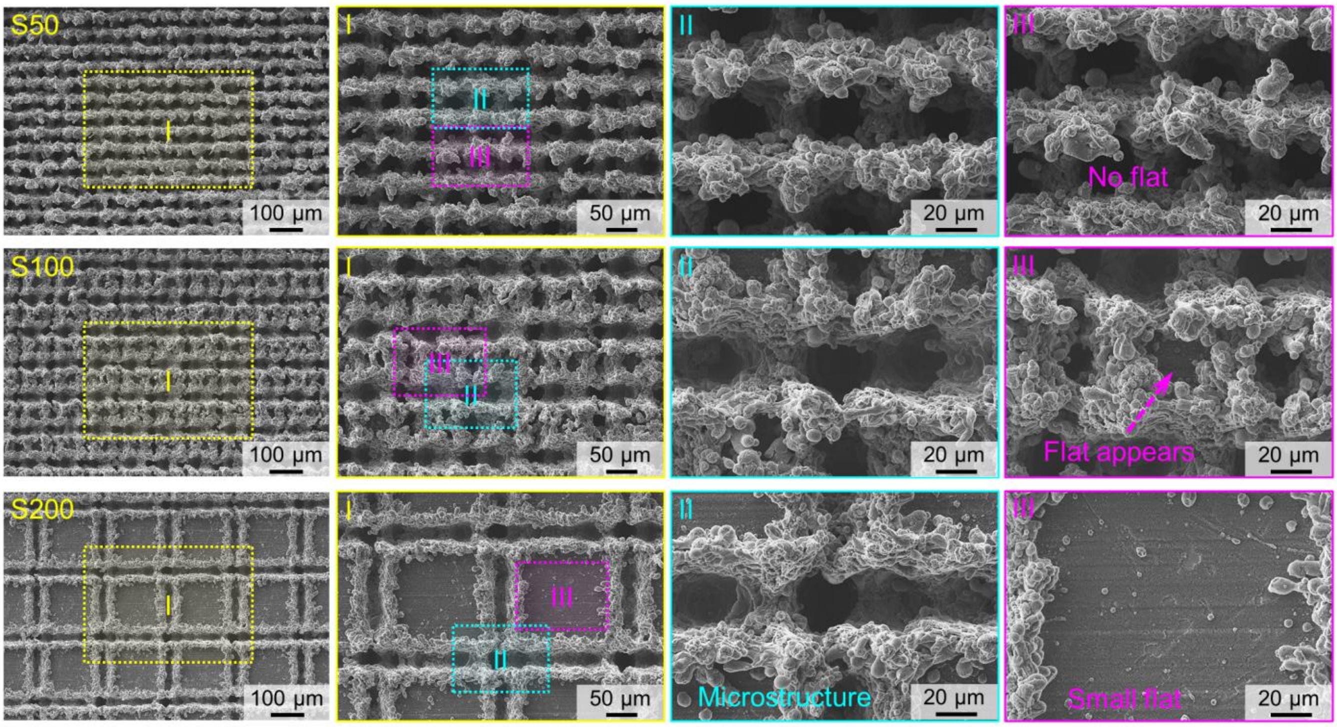

Supplementary Figure 2 | Scanning electron microscope images of laser-ablated microstructures with D_S of 50 μm, 100 μm and 200 μm (pin region). Square-distributed arrays of micro-protrusions were formed on the surfaces due to the grid shape of the laser scanning path. Particles were distributed along laser ablation paths and surrounded each micro-protrusion. There was no flat generated on S50 surface because all areas were ablated, and flats that were not ablated appeared on each micro-protrusion when the laser ablation lines were too sparse to completely cover the surface.

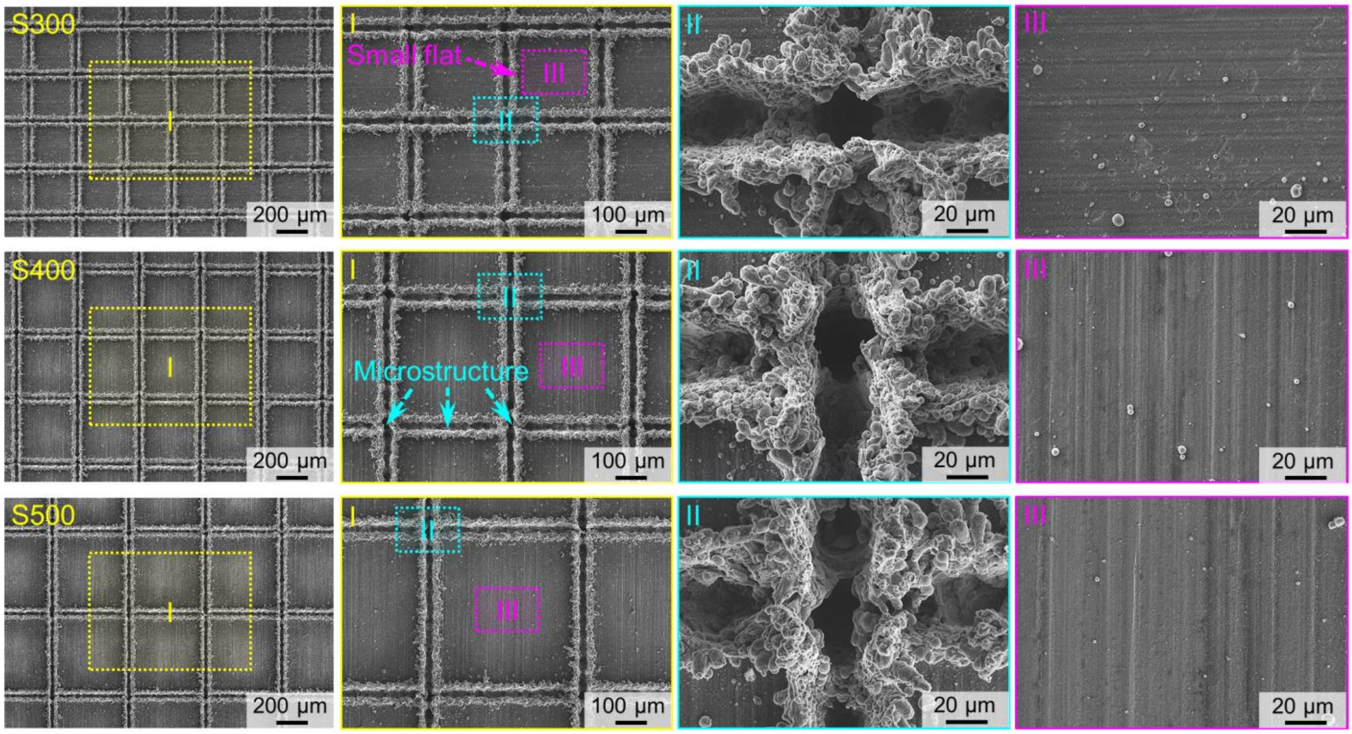

Supplementary Figure 3 | Scanning electron microscope images of laser-ablated microstructures with D_S of 300 μm, 400 μm and 500 μm (gold region). Small flats appeared on micro-protrusions because of the large laser scan spacing, and the flat area became larger as the laser scan spacing continuously increased.

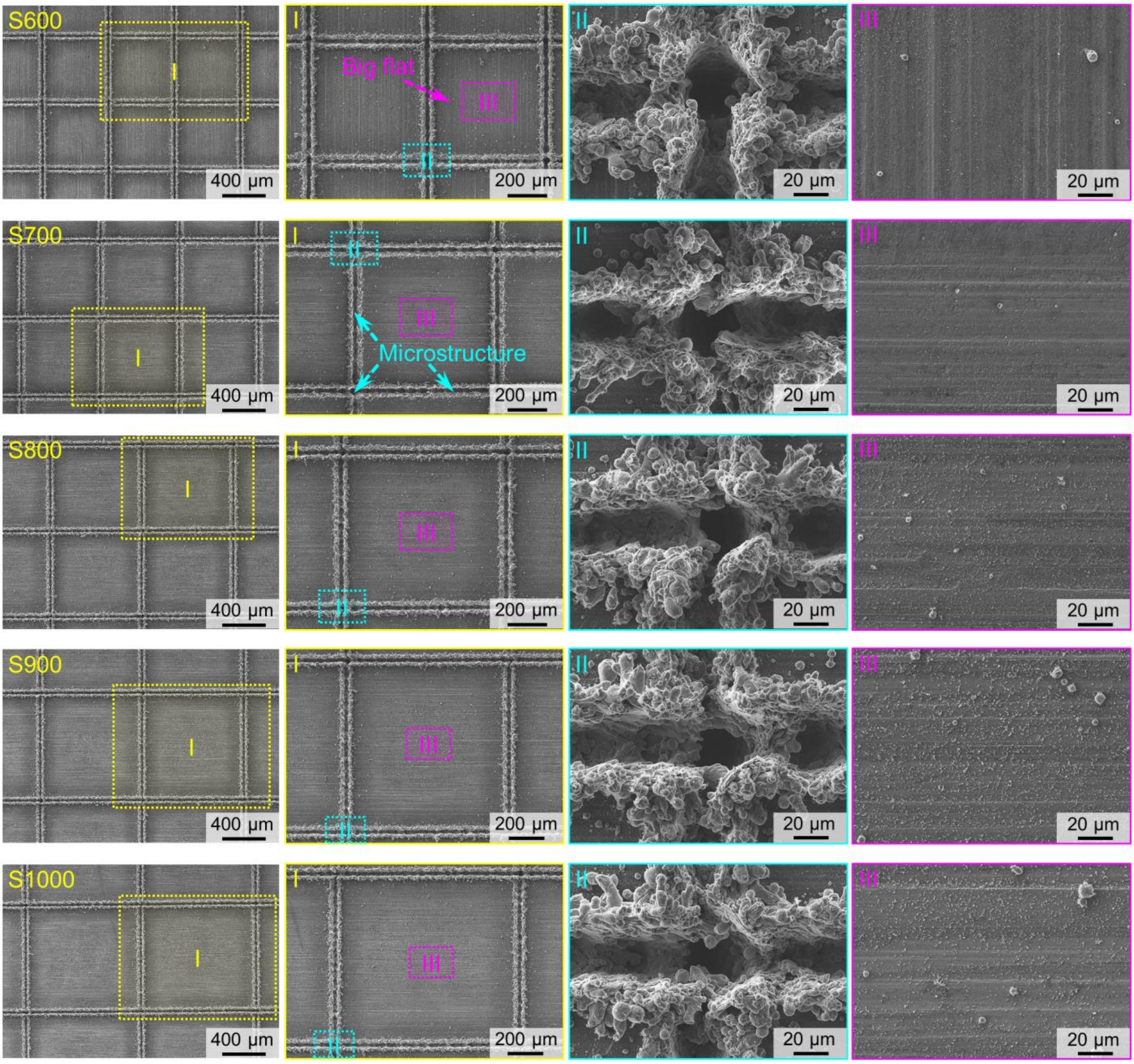

Supplementary Figure 4 | Scanning electron microscope images of laser-ablated microstructures with D_S of 600 μm, 700 μm, 800 μm, 900 μm and 1000 μm (adhesive region). Large flats on micro-protrusions were significant because the laser scan spacing was too large, and the flats were extremely large when the laser scan spacing was set to 1000 μm.

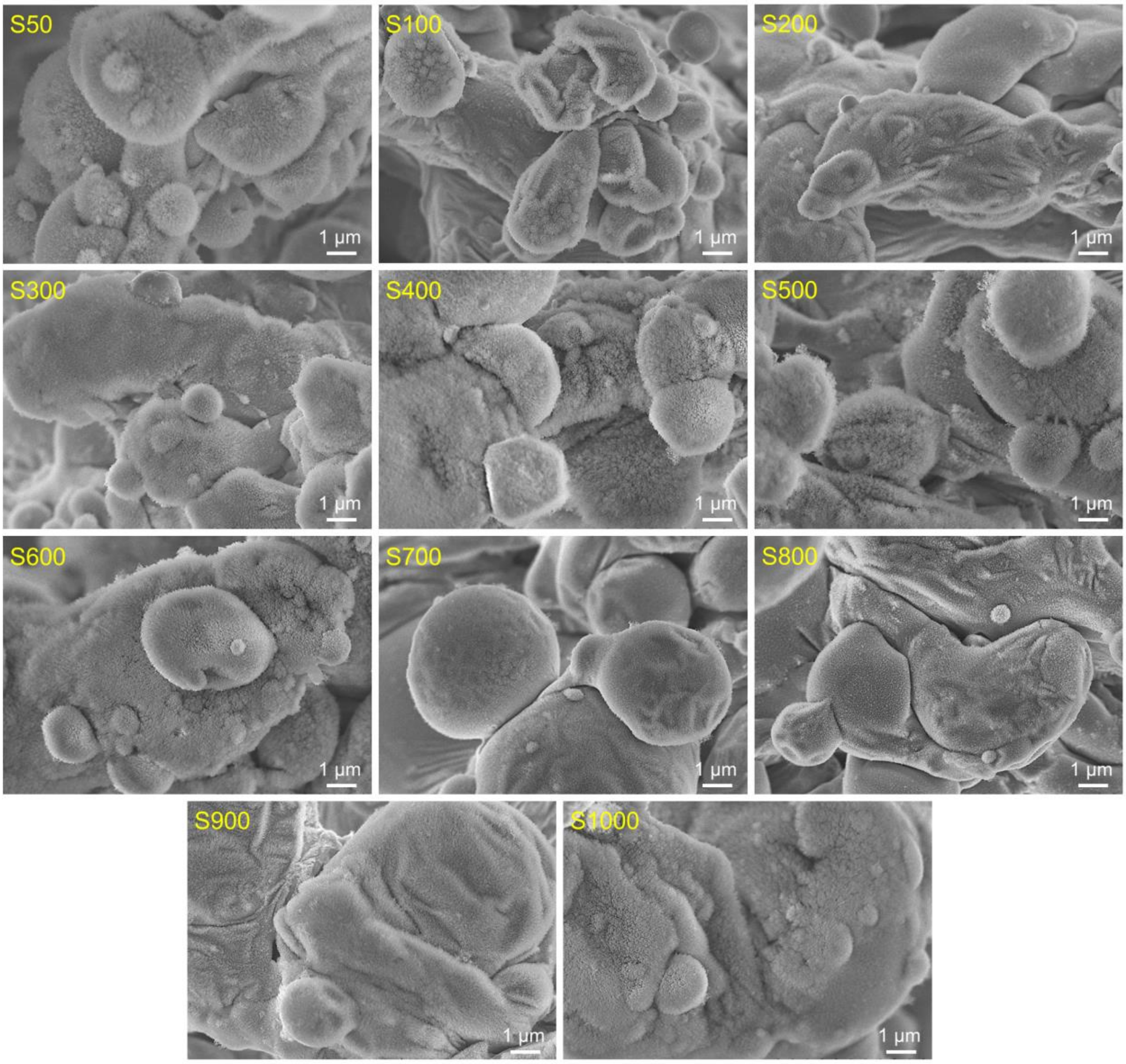

Supplementary Figure 5 | High resolution scanning electron microscope images of laser-ablated microstructures with a series of D_S. The flocculating morphology with scale less than 1 μm was formed due to the cooling of spray materials. No significant difference in morphology corresponding to different laser scan spacings.

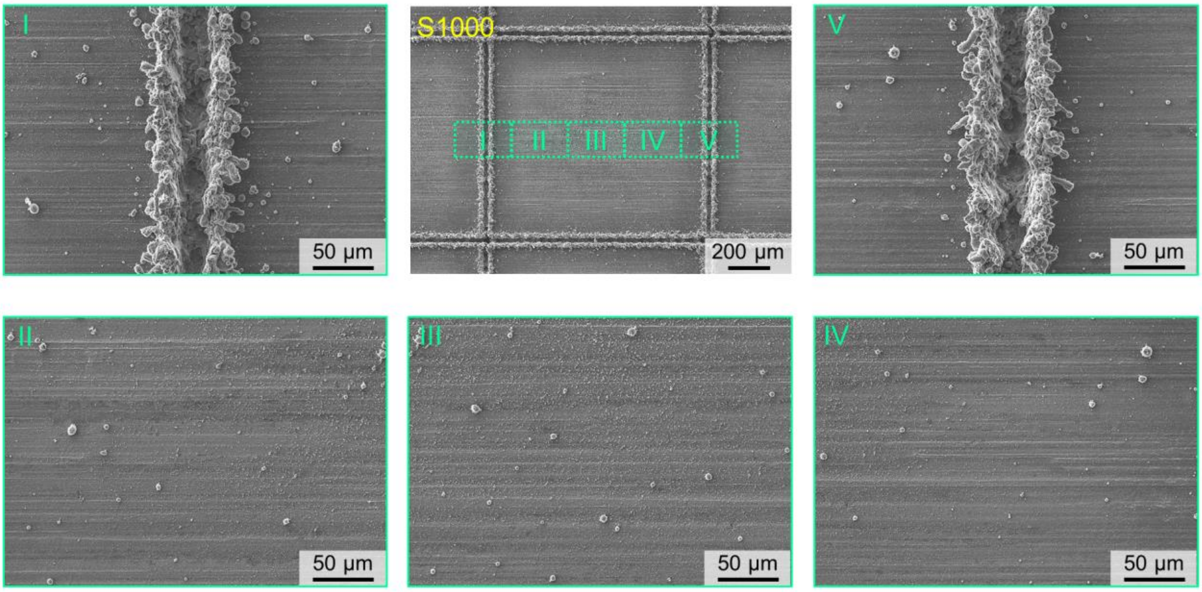

Supplementary Figure 6 | Close-up scanning electron microscope images of the large flat on S1000 surface, showing the morphology near and far from the laser-ablated grooves. Scattered particles were sprayed onto the flats due to the jet effect of laser ablation. However, particles on flats were too sparse to significantly affect morphology and wettability of the surface.

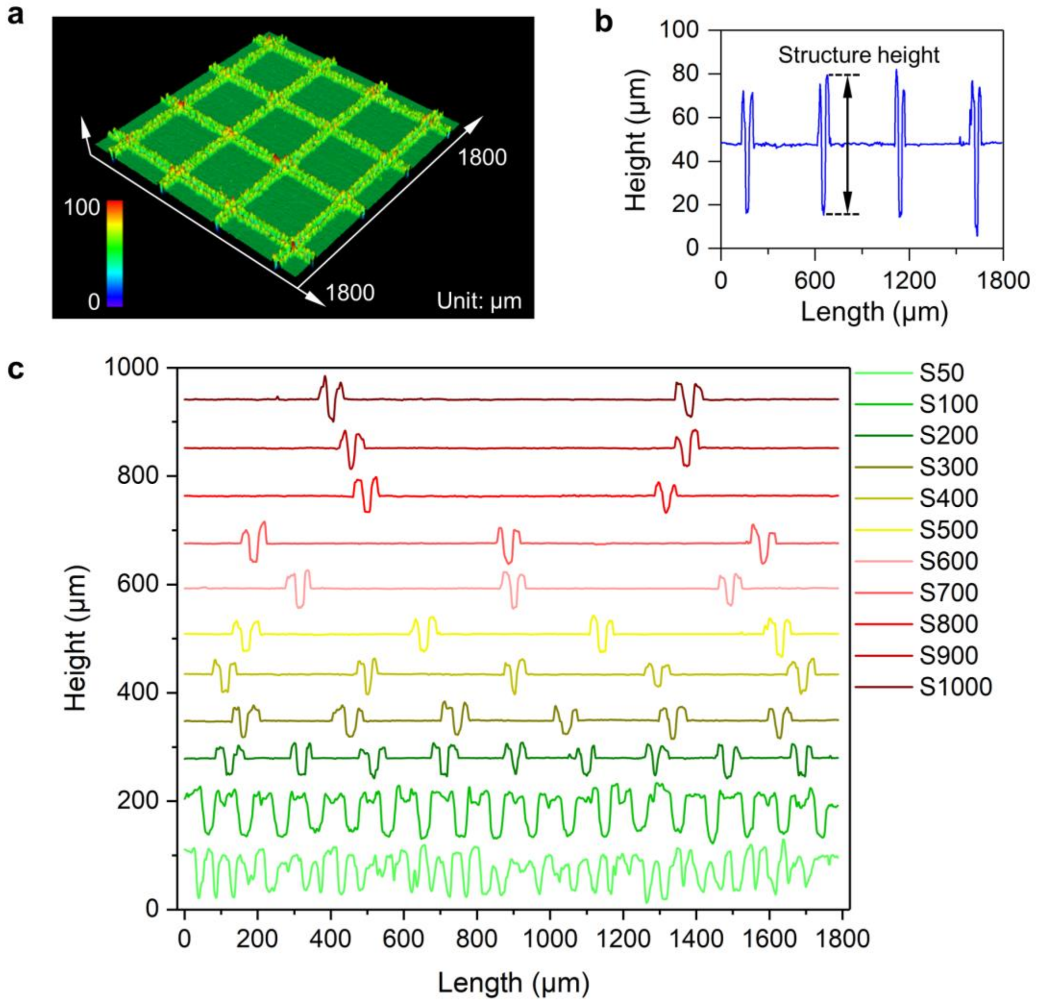

D_S (μm)	50	100	200	300	400	500	600	700	800	900	1000
Average Height (μm)	64.8	82.0	56.6	56.3	57	62.4	63.1	65.2	58.1	64.2	70.3

Supplementary Figure 7 | Geometric scanning of laser-ablated microstructures with a series of D_S. **a-b**, Three-dimensional image and section profile of S500 surface. The D_S was basically consistent with the set laser scanning spacing, and the average height difference between peak and valley of laser ablated grooves was 62.4 μm. **c,** Section profiles of microstructures with D_S of 50-1000 μm. Different laser scan spacings did not significantly change the structure height, and the slight differences in structure heights of different surfaces have almost no effect on the rebound dynamics of droplets.

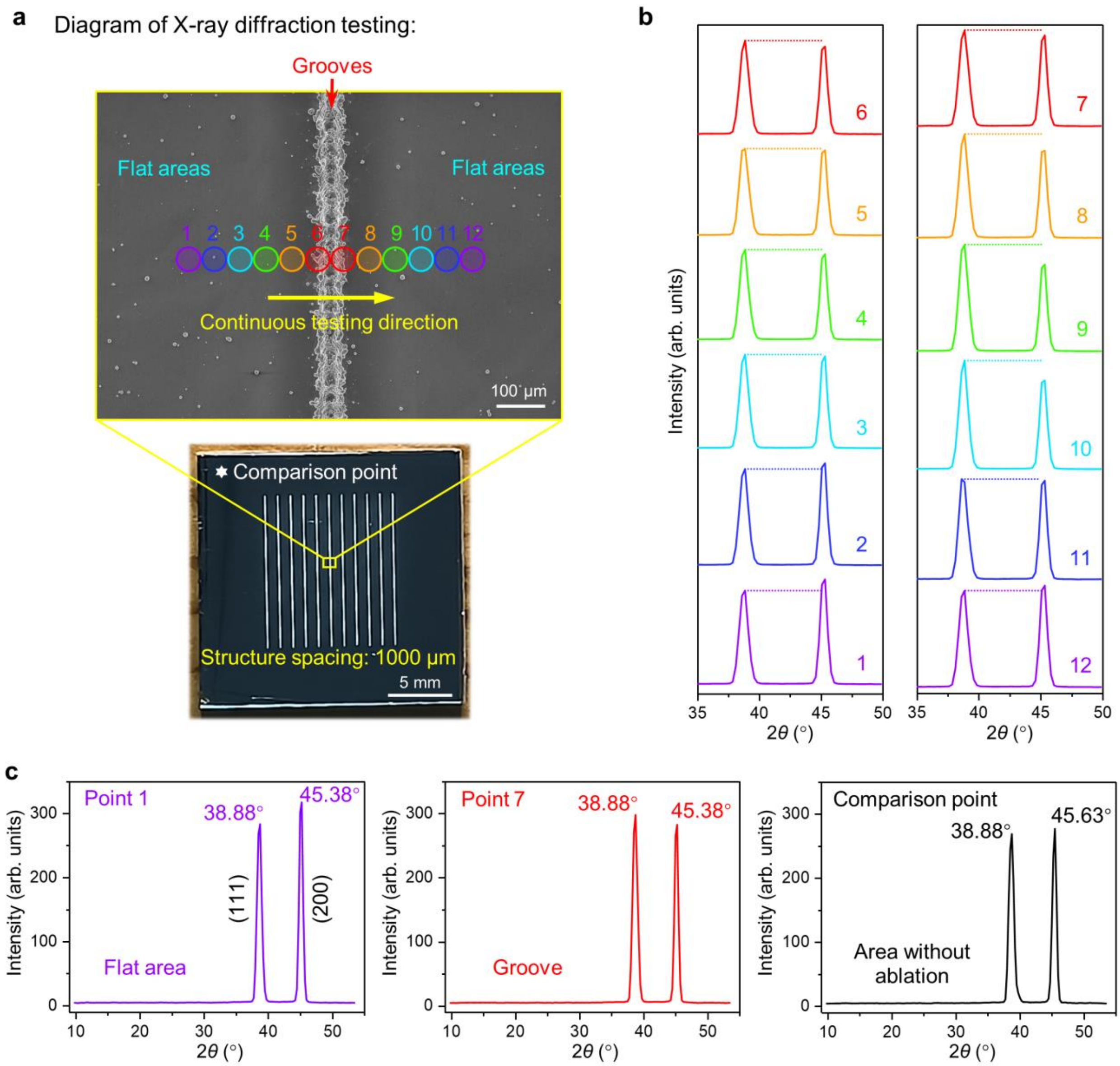

Supplementary Figure 8 | Localized X-ray diffraction results on the laser-scanned grooves and flats. **a**, Diagram of continuous X-ray diffraction scanning across one groove and flats. **b,** The variation of X-ray diffraction peaks with continuous movement of testing position. **c,** Selected X-ray diffraction results of the flat (point 1), the groove (point 7) and the comparison point.

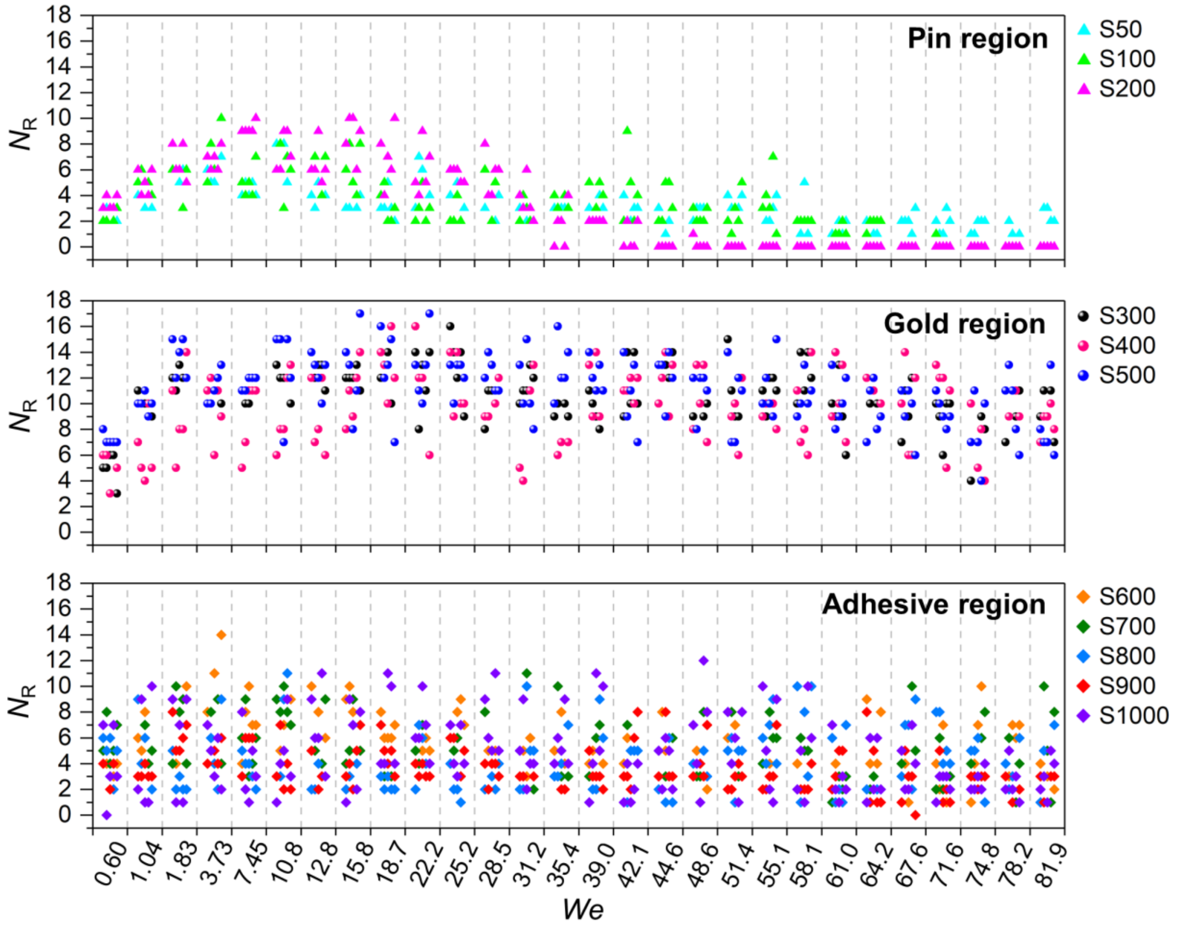

Supplementary Figure 9 | Distribution diagrams of N_R of laser-ablated structures with different D_S at *We* of 0-82. The diagrams have been divided into several blocks corresponding to a series of *We*, and the five points corresponding to the same *We* in each block were dispersed for clear exhibition.

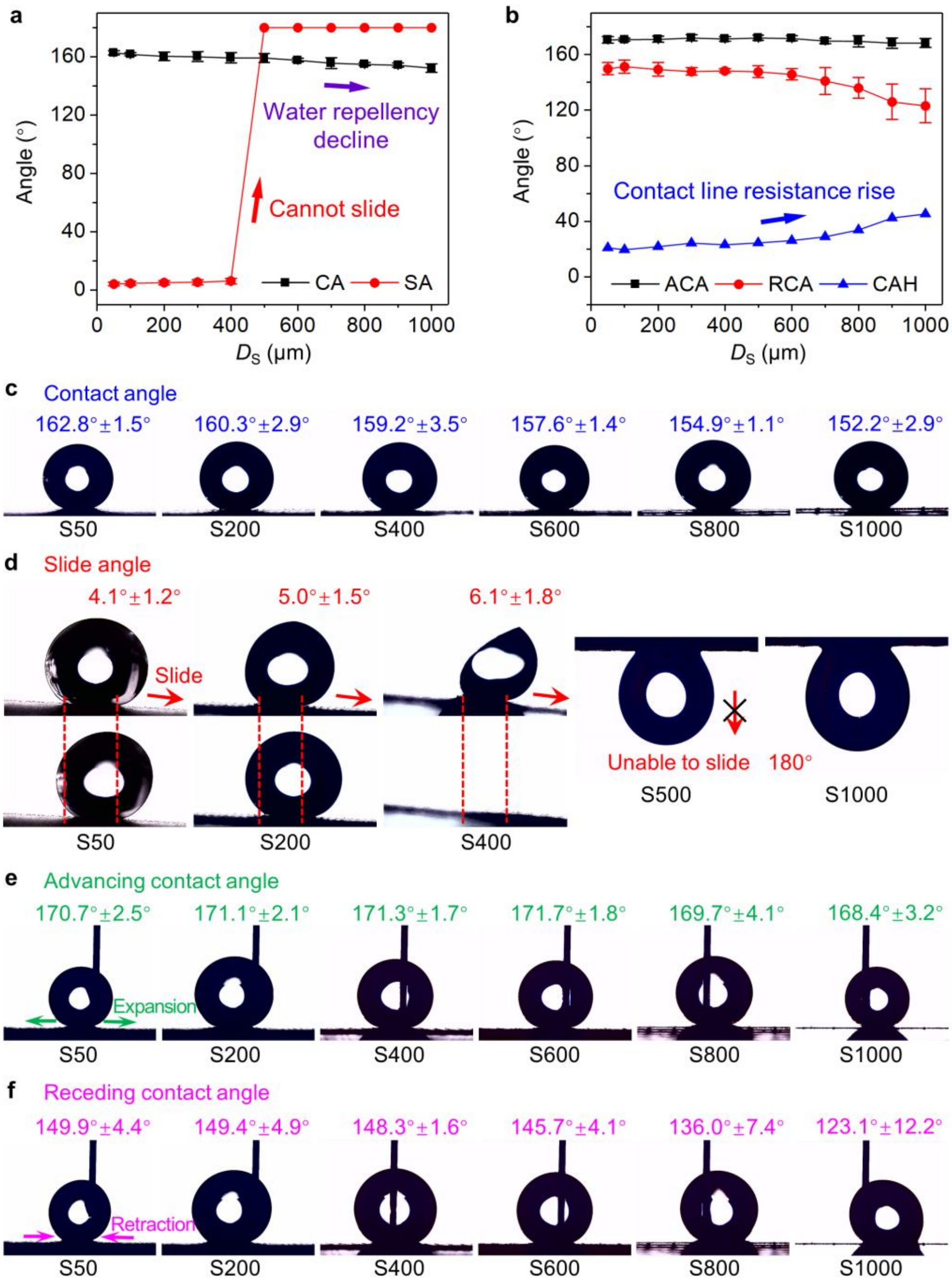

Supplementary Figure 10 | Wettability characteristics of laser-ablated microstructures with different D_S. a, The reduced contact angle (CA) and the increased sliding angle (SA), indicating the weakened water repellency with the continuous increase of D_S. **b,** The advancing contact angle (ACA), receding contact angle (RCA) and calculated contact angle hysteresis (CAH). Large D_S aggravated the heterogeneity of wettability and increased the movement resistance of solid-liquid-air contact lines. **c-f**, Significant supporting images and results for the wettability characterization. All error bars for the angles were obtained from the standard deviation of five replicate experiments.

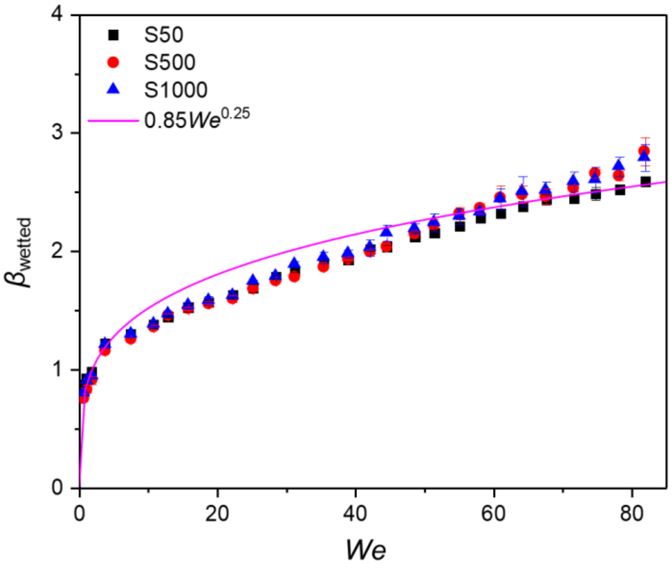

Supplementary Figure 11 | Maximum wetted factors (β_{wetted}) of selected microstructures with three typical D_S as a function of *We*. Experimental factors β_{wetted} of three surfaces were close to $\beta_{wetted}=0.85We^{0.25}$. Droplets were easier to spread on the surfaces of S500 and S1000 and had greater wetting diameters especially for high *We* due to the weakening of pinning resistance. The error bars for the β_{wetted} were obtained from the standard deviation of five replicate experiments.

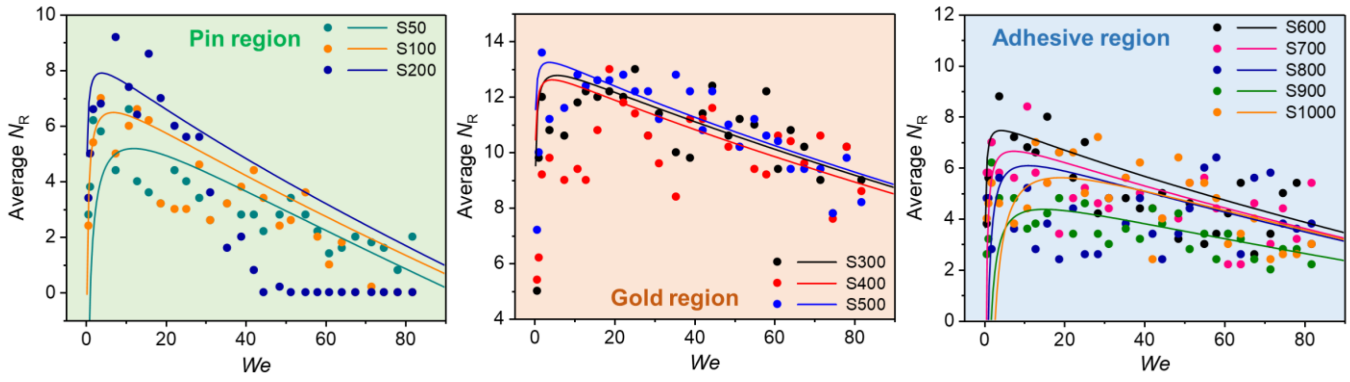

Supplementary Figure 12 | The analysis of the correlation between *We* and N_R. The points originate from the experimental data, and the lines are fitted according to the formula of N_R. Each point is the average value of five points at the corresponding *We* in Supplementary Fig. 9. The fitted factor *A* is 50. The fitted factors *B* of viscous dissipation are 0.03, 0.013 and 0.02, respectively. The coefficients *N* of S50, S100 and S200 in pin region were 10, 9.5 and 10, respectively. The coefficients *N* of S300, S400 and S500 in gold region were 14.5, 14 and 14.5, respectively. The coefficients *N* of S600, S700, S800, S900 and S1000 in adhesive region were 8.5, 8, 7.8, 6 and 8.3, respectively. Especially, the adhesive dissipation only occurs on the surfaces of S600-S1000, and the factor C=1×10^{-5}.

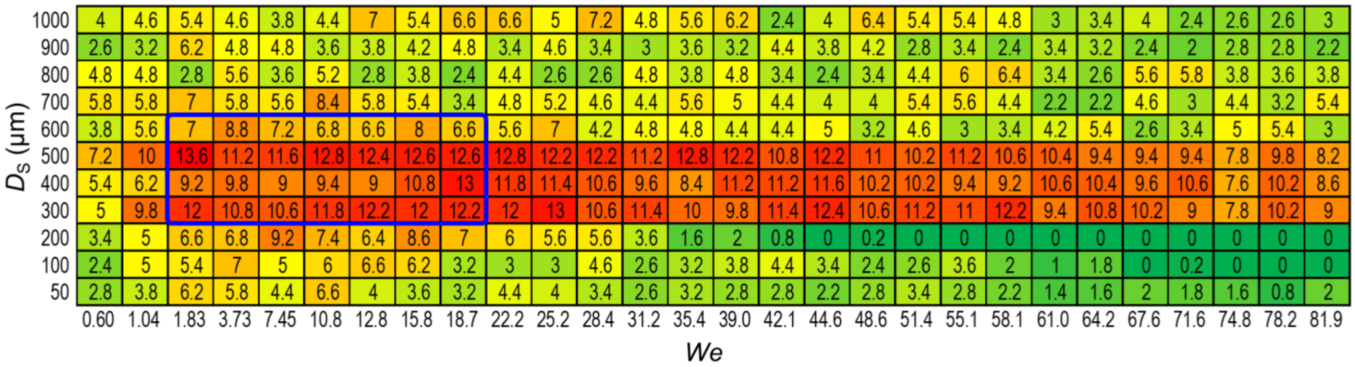

Supplementary Figure 13 | Experimental distribution of average N_R of microstructures with different D_S at different *We*. The marked blue box is the area with high N_R which is similar to the theoretical distribution.

Supplementary Information

S1. The effect of the structure height on the rebound dynamics of droplets

Generally, an impinging droplet first spreads laterally to a maximum diameter, then retracts, and finally detaches from a superhydrophobic surface, showing the conventional bouncing behavior. However, pancake bouncing as a novel droplet rebound behavior has been found on copper substrate with micro-posts, which were fabricated by wire cut electrical discharge machining to achieve large aspect ratio. [1] The large aspect ratio structure can induce pancake bouncing of the droplet, significantly reducing the contact time and promoting the separation of the droplet from the surface when the structure height is large enough. Furthermore, when the structure height is sufficient to allow for adequate capillary energy storage, the emergence of pancake bouncing is rather insensitive to the post height. For much shorter posts, the pancake bouncing cannot be observed owing to insufficient energy storage. Similar results also appeared on aluminum micro-conical pillar arrays fabricated by laser ablation. [2] There exists a critical height of laser-ablated superhydrophobic aluminum structures, and a structure height higher than the critical height can induce the pancake bouncing of droplets. Conventional bouncing processes of the droplet with large contact time were observed when the structure height was smaller than the critical value. With the further increase of the structure height, pancake bouncing of the droplet with the contact time approximately 73% shorter than the conventional contact time occurred on superhydrophobic micro-conical pillar arrays.

High structure can induce the pancake bouncing of droplets, so how does the short structure affect the dynamics of droplet rebounds? Scan speed of the laser beam affects the structure height of laser-ablated aluminum surfaces, and the structure height can be reduced by increasing the scan speed. [3] The rebound behavior of droplet on short structures with the height less than 100 μm is significantly influenced by the scan speed, which directly affects the structure height. The water droplet can rebound from the surface twice due to the extremely low adhesion of the surface when the scan speed is low, and the times of rebound decreases to one as the scan speed rises. Then, the droplet cannot leave the surface as the scan speed continues to increase and it sticks to the surface, which indicates a high adhesion to droplet.

Therefore, high structure can facilitate droplet rebound, and droplets cannot rebound on structures with too small height. Droplet rebound dynamics on shape memory polymer (SMP) pillars with commercial spray (Never Wet) as a non-metallic surface, including the effect of the structure height on droplet rebound behaviors have also been studied. [4] The pancake bouncing was present at the specific range of structure height. However, the droplet shows conventional bouncing when the structure height is larger or smaller than the critical values of the specific range. That means structure height affects the droplet rebound behavior, and pancake bouncing appears only within a specific range of structure heights.

In summary, the structure height can qualitatively affect the droplet rebound behavior, and thus affect the contact time of the droplet. Specific structure height, especially large structure heights, can induce pancake bouncing with significant short contact time of the droplet, and the droplet cannot rebound on structures with too small height. The laser scan speed which could change the structure height was constant in our experiment, and the change in laser scan spacing could not significantly affect the structure height. However, structure height was slightly different in our experiments due to the laser processing technology. But the structure height almost all less than 100 μm, significantly lower than the structure height that induces pancake bouncing of droplet. Meanwhile, pancake bouncing did not occur on our laser-ablated structures, and the structures are high enough to support the rebound of the droplet.

S2. The thermal effect of laser ablation on aluminum grains

We used the 6061 aluminum sheet with a thickness of 0.3mm (thinner sheets may be pierced by the laser according to the height of the laser-ablated structure) in order to minimize the interference of the substrate layer on the X-ray diffraction of the laser-ablated layer. The average grain size of the used 6061 aluminum substrate was 18.25 μm, [5] and the focal size of micro-XRD was about 50 μm. Thus, accurately distinguishing the effect of laser ablation on grain characteristics is difficult. Striped grooves were ablated in a single direction, and flat bars were distributed between the grooves as shown in Supplementary Fig. 8a for accurately identifying the X-ray diffraction results of grooves and flats. Meanwhile, continuous X-ray diffraction along the vertical direction of the laser-ablated grooves as shown in Supplementary Fig. 8a ensured the acquisition of crystallographic features of the groove and

flat.

X-ray diffraction peaks of aluminum appeared at 38.88°, 45.38°, corresponding to the aluminum crystal orientation of 111 and 200, respectively. The intensity of diffraction peaks at 38.88°, 45.38° continuously changed during the continuous testing process, indicating the difference in the crystallographic characteristics between the laser-ablated groove and the flat as shown in Supplementary Fig. 8b. Specifically, the intensity of the diffraction peak at 38.88° continuously increased while the intensity of the diffraction peak at 45.38° continuously decreased, indicating that the thermal effect of the laser induced a transition in crystal orientation. We supplemented confirmatory testing on the edge of the same substrate to verify the intensity of X-ray diffraction peaks corresponding to laser-ablated grooves and the flat between grooves as shown in Supplementary Fig. 8a. The selected testing point was far from the laser ablation area and the same substrate ensured the same original crystal characteristics, avoiding the concentrated thermal effect of the laser and the differences in original crystal characteristics of different substrates, respectively. The temperature rise was limited and could not significantly change the crystal characteristics at the edges of the substrate although the temperature of the substrate also integrally increased during the progress of laser ablation. Therefore, the X-ray diffraction results of grooves and flats could be distinguished through confirmatory testing at the edge of the substrate.

The diffraction peak corresponding to the aluminum crystal orientation of 111 was relatively weaker than the diffraction peak corresponding to the aluminum crystal orientation of 200 for the comparison point as shown in Supplementary Fig. 8c. Specially, the diffraction peak corresponding to the aluminum crystal orientation of 200 appeared at a slightly larger angle of 45.63°, indicating that the laser induced the left shift of the diffraction peak corresponding to the aluminum crystal orientation of 200. Hence, laser ablation induced a transition of the aluminum crystal orientation at grooves from 200 to 111 compared to the flat and the substrate. Similar conclusions of the aluminum crystal orientation can also be found from previous studies. [5] The progress of laser ablation also induced lattice distortion at both grooves and flats in laser-treated area compared to the edges of the substrate.

S3. Formula fitting of N_R based on *We*

We fit the experimental results of average N_R for each surface on the basis of a wide range of *We*, i.e., the correlation between N_R and *We*, to verify the validity of the derived theoretical formula (Supplementary Fig. 12). Both the fitted lines and experimental points of average N_R show a trend of increasing first and then decreasing as *We* increases, indicating that the theoretical model and experimental results are basically consistent. With regard to the fitted lines of S50-S500 surfaces, the maximum value of average N_R increases with larger D_S, attributed to D_S only appearing in the pre-factor of pin dissipation as a positive variable for the surfaces of the pin region and the gold region. The experimental maximum average N_R of S50-500 surfaces are 6.6, 7, 9.2, 13, 13, and 13.6, which are consistent with the increasing trend of the theoretical lines. With regard to the surfaces of the adhesive region, the adhesive dissipation caused by large hydrophilic flats induces lower N_R with the larger D_S, although the pin dissipation is weakened. However, the N_R shows a decreasing trend with fluctuations due to the reduced sensitivity as the D_S increases, which is basically consistent with the trend of theoretical values. Specifically, abnormal phenomenon of N_R at relatively high *We* occurred on S100 and S200 surfaces (especially on the S200 surface). Flat appeared due to the inability of the ablation area to fully cover the surface when the laser scan spacing was larger than 50 μm. Hydrophilic flats on S100 and S200 surfaces facilitated penetration effect and droplet residue, thereby inhibiting the consecutive droplet rebound. Meanwhile, for the S100 and S200 surfaces, the structure spacings were still very small, inducing pin effect. As a result, the S100 and S200 surfaces have two disadvantages: small structure spacing and hydrophilic flat, which are unfavorable for consecutive droplet rebound. The large dynamic pressure brought by relatively high *We* can facilitate penetration effect, which has been confirmed. [6] Therefore, S100 and S200 surfaces exhibited worse consecutive rebound performance of droplets compared to S50 surface at relatively high *We*. However, the numbers of consecutive droplet rebound of S100 and S200 surfaces were still higher than those of S50 surface at relatively low *We*, confirming the weakening of pin effect by increasing structure spacing, as shown in Supplementary Fig. 9.

References

[1] Liu Y. H. et al. Pancake bouncing on superhydrophobic surfaces. Nat. Phys. 10, 515-519 (2014).

[2] Pan, W. H., Wu, S., Huang, L., Song, J. L. Large-area fabrication of superhydrophobic micro-conical pillar arrays on various metallic substrates. Nanoscale 13, 14023-14034 (2021).

[3] Song, Y. X. et al. Controllable superhydrophobic aluminum surfaces with tunable adhesion fabricated by femtosecond laser. Opt. Laser Technol. 102, 25-31 (2018).

[4] Song, J. L. et al. Large-area fabrication of droplet pancake bouncing surface and control of bouncing state. ACS Nano 11, 9259-9267 (2017).

[5] Zhao, S. T. et al. Efficient fabrication of ternary coupling biomimetic superhydrophobic surfaces with superior performance of anti-wetting and self-cleaning by a method. Mater. Des. 223, 111145 (2022).

[6] Lafuma, A., Quere, D. Superhydrophobic states. Nat. Mater. 2, 457-460 (2003).
