# Supplementary material for: Golden section criterion to achieve droplet trampoline effect on metal-based superhydrophobic surface
Source: Nat Commun. 2023 Oct 18;14:6572. doi: 10.1038/s41467-023-42375-3 (PMC10584815; doi:10.1038/s41467-023-42375-3)
Supplement: Supplementary file 3 — Description of Additional Supplementary Files [file 41467_2023_42375_MOESM3_ESM.docx]

**Description of Additional Supplementary Files**

**File name: Supplementary Movie 1**

**Description:** Consecutive droplet rebounds on S50 surface at *We* of 22.2.

**File name: Supplementary Movie 2**

**Description:** Consecutive droplet rebounds on S500 surface at *We* of 22.2.

**File name: Supplementary Movie 3**

**Description:** Consecutive droplet rebounds on S1000 surface at *We* of 22.2.

**File name: Supplementary Movie 4**

**Description:** Consecutive droplet rebounds on S50 surface at *We* of 61.0.

**File name: Supplementary Movie 5**

**Description:** Consecutive droplet rebounds on S500 surface at *We* of 61.0.

**File name: Supplementary Movie 6**

**Description:** Consecutive droplet rebounds on S1000 surface at *We* of 61.0.
